# Trypanocidal effect of the benzyl ester of N-propyl oxamate: a bi-potential prodrug for the treatment of experimental Chagas disease

**DOI:** 10.1186/s40360-015-0010-4

**Published:** 2015-04-22

**Authors:** Carlos Wong-Baeza, Benjamín Nogueda-Torres, Manuel Serna, Sergio Meza-Toledo, Isabel Baeza, Carlos Wong

**Affiliations:** Biochemistry Department, National School of Biological Sciences, National Polytechnic Institute, 11340 Mexico City, Mexico; Parasitology Department, National School of Biological Sciences, National Polytechnic Institute, 11340 Mexico City, Mexico

**Keywords:** *Trypanosoma cruzi*, Prodrug, Chagas disease, N-propyl oxamate benzyl ester, α-hydroxy acid dehydrogenase-isozyme II inhibition

## Abstract

**Background:**

Chagas disease, which is caused by *Trypanosoma cruzi,* is a major health problem in Latin America, and there are currently no drugs for the effective treatment of this disease. The energy metabolism of *T. cruzi* is an attractive target for drug design, and we previously reported that inhibitors of α-hydroxy acid dehydrogenase (HADH)-isozyme II exhibit trypanocidal activity. N-Propyl oxamate (NPOx) is an inhibitor of HADH-isozyme II, and its non-polar ethyl ester (Et-NPOx) is cytotoxic to *T. cruzi.* A new derivative of NPOx has been developed in this study with higher trypanocidal activity, which could be used for the treatment of Chagas disease.

**Methods:**

The benzyl ester of NPOx (B-NPOx) was synthesized and its activity evaluated towards epimastigotes and bloodstream trypomastigotes (*in vitro*), as well as mice infected with *T. cruzi* (*in vivo*). The activity of B-NPOx was also compared with those of Et-NPOx, benznidazole (Bz) and nifurtimox (Nx). NINOA, Miguz, Compostela, Nayarit and INC-5 *T. cruzi* strains were used in this study.

**Results:**

Polar NPOx did not penetrate the parasites and exhibited no trypanocidal activity. In contrast, the hydrophobic ester B-NPOx exhibited trypanocidal activity *in vitro* and *in vivo*. B-NPOx exhibited higher trypanocidal activity than Et-NPOx, Bz and Nx towards all five of the *T. cruzi* strains. The increased activity of B-NPOx was attributed to its hydrolysis inside the parasites to give NPOx and benzyl alcohol, which is an antimicrobial compound with trypanocidal effects. B-NPOx was also effective against two strains of *T. cruzi* that are resistant to Bz and Nx.

**Conclusion:**

B-NPOx exhibited higher *in vitro* (2- to 14.8-fold) and *in vivo* (2.2- to 4.5-fold) trypanocidal activity towards *T. cruzi* than Et-NPOx. B-NPOx also exhibited higher *in vitro* (2- to 24-fold) and *in vivo* (1.9- to 15-fold) trypanocidal activity than Bz and Nx. B-NPOx is more lipophilic than Et-NPOx, allowing for better penetration into *T. cruzi* parasites, where the enzymatic cleavage of B-NPOx would give NPOx and benzyl alcohol, which are potent trypanocidal agents. Taken together with its low toxicity, these results suggest that B-NPOx could be used as a potent prodrug for the treatment of Chagas disease.

## Background

Chagas disease is a parasitic disease caused by *Trypanosoma cruzi (T. cruzi),* which can be transmitted to humans by hematophagous bugs or as a direct consequence of a blood transfusion using infected blood. Chagas disease is endemic in Latin America, where it affects 8–10 million people. Furthermore, Chagas disease causes about 50,000 deaths per year in Latin America, and approximately 25% of the population is at risk of acquiring the disease [1-3]. There are currently very few drugs available for the treatment of Chagas disease, which is therefore considered to be a “neglected” disease. Furthermore, the drugs currently available for the treatment of Chagas disease, including benznidazole (Bz) and nifurtimox (Nx), are only weakly efficacious against this disease and therefore generally fail to restore patients to full health [4]. The acute phase of the disease appears shortly after infection, and the chronic phase appears after a silent asymptomatic period that can last for several years. The heart, esophagus, colon and peripheral nervous system can be irreversibly damaged during the chronic phase of Chagas disease, and the majority of patients usually die from heart failure [5,6].

Although Bz and Nx can be effective treatments during the acute phase of Chagas disease, they are not effective against every known strain of *T. cruzi* [7]. Nx has been reported to exert its activity via the induction of oxidative stress [8], whereas Bz exerts its activity by producing DNA damage, as well as inhibiting protein synthesis and the respiratory chain [9]. It was recently shown that a trypanosomal type I nitroreductase plays a key role in the activation of Bz and Nx via an oxygen-insensitive pathway, which leads to the formation of a series of highly cytotoxic metabolites, including glyoxal and several nitriles. These types of cytotoxic metabolites can readily react with a variety of other biological molecules to form adducts, which could account for the pleiotropic effects observed in trypanosomes treated with these prodrugs [10,11]. The weak effects of Bz and Nx during the chronic phase of Chagas disease can be attributed to their relatively short half-lives, as well as their poor permeability properties, which afford them limited tissue penetration [12]. Based on these limitations, there is currently a lack of effective drugs for the treatment of Chagas disease, and several new approaches have been developed in an attempt to identify novel drugs that target specific metabolic pathways that are critical to the survival of *T. cruzi* [13].

Inhibitors of ergosterol biosynthesis, such as posaconazole and albaconazole, are highly effective for the treatment of fungal diseases, and several compounds belonging to this particular class have also been shown to exhibit activity against *T. cruzi,* making them suitable candidates for clinical use [14]. K-777 is an inhibitor of cruzipain, which is a cysteine protease that blocks the proliferation of *T. cruzi* [15]. Unfortunately, however, the clinical use of K-777 as a therapeutic agent for the treatment for Chagas disease has been limited by its hepatotoxicity. Inhibitors of pyrophosphate metabolism have multiple enzymatic targets, including squalene synthase, farnesyl pyrophosphate synthase, proton-pumping pyrophosphatase [12] and hexokinase [16], and compounds belonging to this class have been reported to exhibit activity against *T. cruzi,* although their clinical use is still under investigation. Inhibitors of trypanothione have been reported to show trypanocidal activity *in vitro*, but there is currently no evidence to suggest that they can behave in a selective manner *in vivo* [17]. Zyloprim (allopurinol), which is an inhibitor of purine metabolism that is currently used for the treatment of gout in humans, has been shown to exhibit activity towards trypanosomatides, although the activity of this compound towards *T. cruzi* in humans has been the subject of some controversy, and several other purine metabolism inhibitors are currently being evaluated [18].

Glycolysis has been identified as the only ATP-generating process in *T. cruzi* parasites [19], and it was consequently envisaged that the treatment of these organisms with inhibitors capable of reducing their level of glycolytic flux would result in their death [20]. *T. cruzi* has an NAD-linked α-hydroxy acid dehydrogenase (HADH) that is similar to the lactate dehydrogenase X isozyme of mammalian spermatozoa, which has two isozymes. Isozyme I of this enzyme is responsible for the weak lactate dehydrogenase activity found in *T. cruzi*, whereas isozyme II is active towards α-ketocaproate and α-ketoisocaproate. Furthermore, because isozyme II is involved in the oxidation of NADH to NAD^+^ during glycolysis, it plays an important role in the pathways that supply energy to *T. cruzi* [21]. α-Ketocaproate and α-ketoisocaproate, which are derived from leucine and isoleucine by transamination, also participate in the NADH shuttle system that transfers reducing equivalents from the cytosol to the mitochondria [22]. Inhibitors that block the activity of HADH-isozyme II could therefore be used to inhibit both glycolysis and the NADH shuttle system [23-26], which would lead a significant decrease in the energy being supplied to *T. cruzi* and the death of the parasite.

We have shown that N-allyl oxamate (NAOx), N-propyl oxamate (NPOx) and N-isopropyl oxamate (NIPOx) are selective inhibitors of HADH-isozyme II, based on the chemical structures of the compounds being similar to those of the isozyme II substrates, α-ketocaproate and α-ketoisocaproate. Although these oxamates were unable to penetrate intact *T. cruzi*, the corresponding hydrophobic ethyl esters (i.e., Et-NAOx, Et-NPOx and Et-NIPOx) successfully penetrated intact parasites and exerted trypanocidal activity [27,28]. Et-NAOx, Et-NPOx and Et-NIPOx also exhibited inhibitory activity towards amastigote nests [29]. It was envisaged that the non-specific aliphatic and aromatic carboxyl esterases found in *T. cruzi* [30,31] would hydrolyze these esters after they had penetrated the *T. cruzi* and release the inhibitors, which would then block the activity of HADH-isozyme II and kill the *T. cruzi*. In this context, the esters of NPOx would be behaving as prodrugs, in the sense that they must undergo a metabolic conversion process before becoming an active pharmacological drug [32].

In this study, we have investigated the development of an efficient prodrug for the treatment of Chagas disease by esterifying NPOx with a compound more hydrophobic than ethyl alcohol. Benzyl alcohol was selected because as an appropriate alcohol for the esterification of NPOx not only because of its high hydrophobicity, but also because it is a well-known antimicrobial agent [33]. The high lipophilicity of the benzyl ester of NPOx (B-NPOx) would allow it enter intact parasites more effectively, and its subsequent enzymatic cleavage would result in the release the inhibitor NPOx and benzyl alcohol, which would exert a dual trypanocidal effect on *T. cruzi* parasites. B-NPOx was synthesized and characterized as part of the current study, and its activity was evaluated on *T. cruzi* epimastigotes and trypomastigotes (*in vitro*), as well as the blood and tissues of *T. cruzi* infected mice (*in vivo*), using five *T. cruzi* strains. The activity of B-NPOx was also compared with those of the reference drugs Bz and Nx.

## Methods

### Chemicals

NAD, NADH, pyruvate, α-ketoisocaproate sodium salt and N-ethylmaleimide were obtained from Sigma Chemical Co. (St. Louis, MO, USA). Nifurtimox was purchased from Bayer (Munich, Germany) and benznidazole was from Roche (Basel, Switzerland). NPOx and Et-NPOx were synthesized according to previously reported methods [24]. All of the chemicals used in the current study were of the highest purity available.

### Synthesis of the benzyl ester of N-propyl oxamate (B-NPOx)

A solution of propylamine (0.1 mol) in ether (50 mL) was added in a dropwise manner to an ice-cold solution of dibenzyl oxalate (0.1 mol) in a 4:1 (v/v) mixture of diethyl ether and chloroform (250 mL), and the resulting mixture was stirred for 2 h at 5°C. The reaction mixture was then warmed to room temperature and stirred overnight. The benzyl alcohol produced during the reaction was subsequently removed by distillation at 50–60°C/1 mmHg to give a cloudy residue, which was dissolved in diethyl ether (10 mL) and held for 12 h at 5°C. A crystalline product formed with a melting point of 64–65°C, which was collected by filtration. The filtrate was evaporated to dryness under reduced pressure and then fractionated under vacuum. B-NPOx distilled at 60–65°C/1 mmHg as a colorless oil, which crystallized on standing. The crystals were purified by recrystallization from chloroform to give the desired product in an overall yield of 86% with a melting point of 50–55°C. The final product was analyzed by IR, ^1^H-NMR, ^13^C-NMR and HRMS. IR KBr (Perkin Elmer GX spectrometer; Perkin Elmer, Waltham, MA, USA): 3292 (N-H), 3036, 2966 (C-H, aromatic), 1785 (C = O), 1514 (C = C), 1377 (C-H methyl), 1222 (C-N-C), 898 (C-O-C), 765, 895 (aromatic, monosubstituted) cm^-1^. ^1^H-NMR (Jeol JNM-GSX270 spectrometer; Jeol, Pleasanton, CA, USA): (270 MHz, CDCl_3_) δ 0.91 (t, *J =* 7.4 Hz, 3H, CH_3_), 1.53 (s, 2H, CH_2_), 3.25 (q, *J =* 7.2 Hz, 2H, CH_2_), 5.29 (s, 2H, CH_2_), 7.1 (bs, 1H, NH), 7.32–7.37 (m, 5H, H phenyl). ^13^C-NMR (Jeol JNM-GSX270): (67.5 MHz, CDCl_3_) δ 11.37 (CH_3_), 22.46 (CH_2_Me), 41.69 (CH_2_-NH), 68.64 (CH_2_-O), 128.72 (C_o,m_ phenyl), 128.87 (C_p_ phenyl), 134.47 (C_ipso_ phenyl), 156.38 (CO-NH), 160.75 (COO). HRMS (Bruker micrOTOF-Q II 10392 spectrometer; Bruker, BioSpin Co. Billerica, MA, USA): Calcd for C_12_H_15_NO_3_: *m/z* = 221.2524 [M]^+^; found *m/z* = 221.1085 [M]^+^. These results confirmed that our synthesis of B-NPOx was successful.

### Determination of the cytotoxicity of NPOx, Et-NPOx and B-NPOx

The cytotoxic activities of NPOx, Et-NPOx and B-NPOx were evaluated against monkey kidney epithelial cells (Vero cell line) using an alamar blue (Invitrogen, Life Technologies, Sacramento, CA. USA) viability assay. The cells were cultured in standard culture medium (MEM with 10% fetal bovine serum) in 24-well plates (7.5 × 10^3^ cells/well) to 80% confluence. Increasing concentrations of NPOx, Et-NPOx and B-NPOx (0.1–3 mM) in 5 μL of ethyl alcohol were added to each well. The medium was replaced after 24, 48 and 72 h of incubation (37°C, 5% CO_2_ and 95% relative humidity) with 200 μL of fresh medium containing alamar blue, and the fluorescence of each well was measured using a Perkin Elmer LS 55 spectrofluorometer (Perking Elmer, Waltham, MA, USA). Cell viability (%) was measured relative to the control wells (cultured in medium alone) according to the following calculation: Cell viability (%) = (fluorescence_test_ × 100%)/fluorescence_control_.

### Determination of the toxicity of Et-NPOx and B-NPOx on mice

The median lethal dose (LD_50_) values of Et-NPOx and B-NPOx were determined in NIH male albino mice (18–20 g), according to the established requirements [34]. Et-NPOx and B-NPOx were orally administered as solutions in a 5% Arabic gum in water at doses of 1.5–3.0 g/kg of body weight. The drugs were administered on a daily basis for 50 days.

### *Trypanosoma cruzi* strains

The *T. cruzi* strains used in the current study were isolated from triatomines and chagasic patients. The Parra and Compostela strains of *T. cruzi* were isolated from *Triatoma longipennis*. The Nayarit strain of *T. cruzi* was isolated from *Triatoma picturata*, and the NINOA (MHOM/MX/1994/Ninoa) and INC-5 (MHOM/MX/1994/INC-5) strains were isolated from chronic chagasic patients living in two endemic areas of Mexico [35]. The NINOA and INC-5 strains correspond to the prevalent discrete typing unit (DTU) classification designated as TcI, and it is also likely that the Parra, Compostela and Nayarit strains belong to the same TcI classification [36] as the other strains of Mexican *T. cruzi* characterized to date [35]. Following their isolation, the strains were maintained in triatomine bugs *(Meccus longipennis)* and by serial passages in NIH male albino mice [37].

### Culture of *Trypanosoma cruzi* epimastigotes

Cardiac blood samples from NIH male albino mice that had been individually infected with the different *T. cruzi* strains were cultured at 28°C in liver infusion tryptose (LIT) broth supplemented with 10% heat-inactivated fetal calf serum [38]. When the growth of the epimastigotes became exponential, they were collected from the liquid phase by centrifugation at 3000 × g for 15 min. The resulting pellet was then washed three times with 20 volumes (with respect to the pellet) of buffer containing 0.1 M sodium phosphate (NaH_2_PO_4_/Na_2_HPO_4_) and 0.15 M NaCl (pH 7.4) and resuspended in the same buffer at 1 × 10^6^ epimastigotes/mL. This suspension was then aliquoted (1 mL) in Eppendorf tubes. All of these procedures were carried out at 4°C.

### *Trypanosoma cruzi* homogenates and enzyme preparations

One milliliter samples of the *T. cruzi* suspensions described above were subjected to three cycles of freezing in liquid nitrogen and thawing at room temperature. The disruption of the parasites was monitored by the microscopic examination of the resulting homogenate, which was frozen at –20°C until it was required for the experiments. There was no discernible decrease in the HADH-isozyme II activity of the homogenates after 2 months in storage at –20°C. The homogenates were thawed at room temperature immediately prior to be used in the enzymatic assays, and centrifuged at 1200 × g for 20 min at 4°C. The supernatant of each homogenate (enzyme preparation) was used to analyze the HADH-isozyme II activity. HADH-isozyme II was purified as previously described [21]. The homogenates were prepared without any protease inhibitor, which could inhibit the aliphatic and aromatic carboxyl esterase activities described in *T. cruzi* [30,31].

### Quantification of α-hydroxy acid dehydrogenase isozyme II activity

HADH-isozyme II activity was measured spectrophotometrically by following the oxidation of NADH (λ = 340 nm, ε = 6220 M^-1^ cm^-1^) with α-ketoisocaproate as a substrate [21]. Each assay mixture contained, in a final volume of 3 mL, 0.12 mM NADH, 0.1 M sodium phosphate (NaH_2_PO_4_/Na_2_HPO_4_) plus 0.15 M NaCl (pH 7.4) buffer, 5 mM neutral sodium salt of α-ketoisocaproate, and the purified enzyme or an enzyme preparation from the homogenate of each one of the five *T. cruzi* strains. The purified enzyme or enzyme preparation was diluted with the 0.1 M sodium phosphate plus 0.15 M NaCl (pH 7.4) buffer prior to being used to allow for a shift in the absorbance at 340 nm of 0.05–0.08 nm/min with 5 mM α-ketoisocaproate. The assay mixtures were incubated at 37°C either alone or in the presence of NPOx, Et-NPOx or B-NPOx, and changes in the absorption at 340 nm were recorded over a 5 min period. In some cases, 1 mM of N-ethylmaleimide, which is a known inhibitor of *T. cruzi* carboxylesterases [31], was added to the assay mixtures. The activity of HADH-isozyme II was subsequently expressed as the percentage of ΔE 340 nm/min, where the velocity in the absence of an inhibitor was considered to be 100%. The results were then plotted against the concentrations of NPOx, Et-NPOx or B-NPOx (0.05–1 mM).

### Trypanocidal activity of B-NPOx towards cultured epimastigotes

Epimastigotes (1 × 10^6^ units/mL) of the five *T. cruzi* strains in the 0.1 M sodium phosphate plus 0.15 M NaCl (pH 7.4) buffer were used to evaluate the trypanocidal activity of B-NPOx. Nine hundred and eighty microliters of the epimastigote suspension was placed in an Eppendorf tube, followed by 20 μL of ethyl alcohol or benzyl alcohol or an ethyl alcohol solution of NPOx, Et-NPOx, B-NPOx, Nx or Bz, at a final concentration in the range of 0.1–3 mM. After being incubated at 28°C for 10, 20, 30, 40, 50 and 60 min, the number of live parasites was determined in a Neubauer haemocytometer [39] using the trypan blue dye exclusion method. The mean number of live epimastigotes in the control group, which was not subjected to any drug treatment, was taken as 100%. The results were subsequently given as the mean values ± the standard deviations of three independent experiments for the viability of the epimastigotes [24].

To measure the penetration of B-NPOx into the epimastigotes, a suspension of epimastigotes (1 × 10^6^ units/mL) in 980 μL of the 0.1 M sodium phosphate plus 0.15 M NaCl (pH 7.4) buffer was mixed with 20 μL of the 1 mM solution of B-NPOx in ethyl alcohol. After being incubated at 28°C for 15, 30 and 45 min, the samples were centrifuged at 3000 × g and the supernatants were analyzed spectrophotometrically at 248 nm, which corresponds to the absorbance peak of B-NPOx. A decrease in the absorbance of the supernatants at 248 nm correlated well with the penetration of B-NPOx into the epimastigotes.

### Trypanocidal activity of B-NPOx on bloodstream trypomastigotes

Bloodstream trypomastigotes of each one of the five *T. cruzi* strains were obtained by cardiac puncture from NIH male albino mice at the peak of parasitaemia. One hundred and ninety-five microlitre aliquots of blood containing 1 × 10^6^ trypomastigotes were added to each well of a sterile 96-well plate, followed by 5 μL of NPOx, Et-NPOx, B-NPOx, Nx or Bz at final concentrations in the range of 0.1–3 mM. Stock solutions of NPOx, Et-NPOx, B-NPOx and the reference drugs in DMSO were prepared and subsequently diluted with sterile distilled water to give a final DMSO concentration of 1%. Blood containing 1 × 10^6^ trypomastigotes with 1% DMSO was used as a control. The plates were incubated at 4°C for 24 h and the trypanocidal effects of the compounds were determined by comparing the motilities of the trypomastigotes at the different concentrations with the control. The results were subsequently presented as the mean values ± the standard deviations of the number of mobile (viable) trypomastigotes from three independent experiments.

### Effect of B-NPOx on the parasitaemia and amastigote nests in infected mice

NIH male albino mice (18–20 g) were inoculated intraperitoneally with 1 × 10^3^ bloodstream trypomastigotes of each of the five different *T. cruzi* strains. The mice were divided in five groups (10 mice per group), including (1) those without any treatment (control group, infected/untreated), and those treated with (2) Bz, (3) Nx, (4) Et-NPOx or (5) B-NPOx. The four test compounds were orally administered as solutions in 5% Arabic gum in water at a dose of 100 mg/Kg [7] per day over a period of 50 days. The first dose was given 24 h after the infection. Levels of parasitemia, beginning 24 h after infection, were determined every other day in a Neubauer hemocytometer using 5 μL of blood, which was collected from the tail vein of the infected mice and diluted at a ratio of 1:10 (v/v) with a saturated solution of ammonium chloride [39]. The reduction in the parasitemia was evaluated by comparing the number of trypomastigotes obtained at each time point after the administration of NPOx, Et-NPOx, B-NPOx, Bz or Nx with the number of trypomastigotes obtained at the same point in infected/untreated mice, which was taken as 100% [29]. The experimental protocols for animal care and use were reviewed and approved by the Bioethics Committee of our Institution according to the “Guide for the care and use of laboratory animals”, which was published by the US National Institute of Health [40].

To evaluate the activity of B-NPOx towards amastigote nests, we obtained tissue samples from the hearts and left legs (skeletal muscle) of mice from each of the five groups described above either 30 or 50 days after infection. The tissue samples were fixed with formaldehyde, dehydrated and embedded in paraffin. Sections of the samples (3 μm thick) were then stained with hematoxylin and eosin and analyzed by light microscopy. Fifty randomly selected microscopic fields from at least three mice were examined to quantify the number of amastigote nests. The mean number of amastigote nests in the infected/untreated group was taken as 100% [29].

## Results

### B-NPOx inhibits the activity of α-hydroxy acid dehydrogenase-isozyme II in homogenates from T. cruzi strains

**Figure 1:**
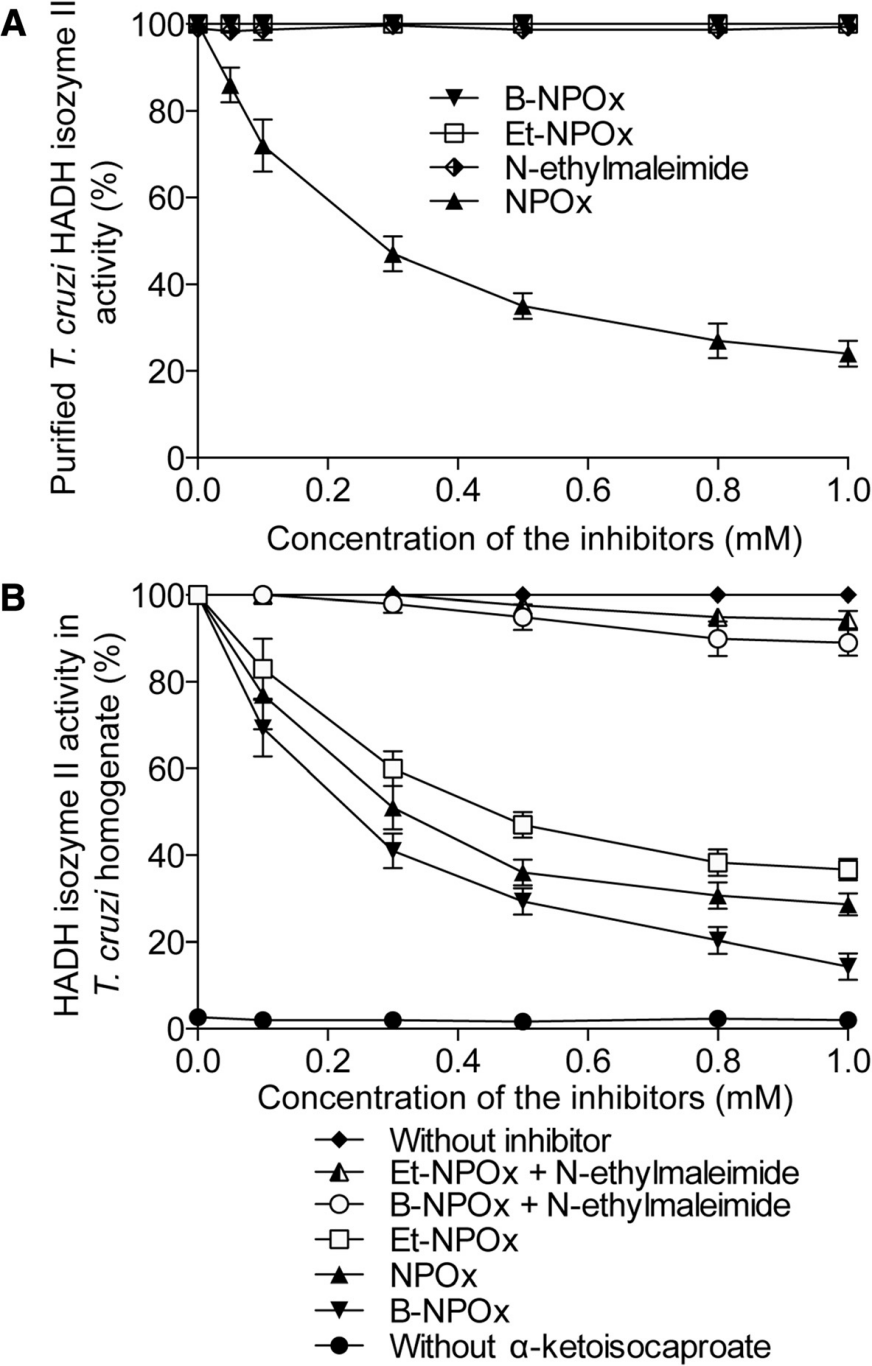
B-NPOx did not inhibit the activity of purified HADH-isozyme II but did exhibit an inhibitory effect towards *T. cruzi* homogenates. **(A)** Effects of B-NPOx, Et-NPOx, NPOx and N-ethylmaleimide (0.05–1 mM) on the activity of purified HADH-isozyme II in the presence of 5 mM α-ketoisocaproate and 0.12 mM NADH. Changes in the absorption at 340 nm were recorded over a 5 min period. The enzymatic activity was expressed as a percentage of ΔE 340 nm/min, where Vmax was 100%. **(B)** Effects of B-NPOx, B-NPOx + N-ethylmaleimide, Et-NPOx, Et-NPOx + N-ethylmaleimide and NPOx (0.05–1 mM) on the enzymatic activities of the *T. cruzi* homogenates. The N-ethylmaleimide concentration was 1 mM and the reaction conditions were set as in **(A)**. As controls, the effects of B-NPOx, Et-NPOx and NPOx on the *T. cruzi* homogenates were assayed in the absence of α-ketoisocaproate. The kinetic assays have been reported as the mean values ± the standard deviation from three independent experiments.

B-NPOx did not inhibit the activity of purified HADH-isozyme II (Figure 1A), and the same effect was reported previously for Et-NPOx [24]. However, B-NPOx did effectively inhibit the activity of the homogenates derived from the NINOA strain of *T. cruzi*, and its inhibitory activity was higher than those of NPOx and Et-NPOx (Figure 1B). When the *T. cruzi* homogenates were incubated with N-ethylmaleimide, which is an inhibitor of *T. cruzi* carboxylesterases [31], prior to being treated with B-NPOx or Et-NPOx, the inhibitory activity of these compounds decreased (Figure 1B). However, the activity of purified HADH-isozyme II was unaffected by the presence of N-ethylmaleimide (Figure 1A). Notably, NADH was not oxidized when α-ketoisocaproate was not added to the homogenates (Figure 1B). Similar results were obtained with the other four *T. cruzi* strains (data not shown).

### B-NPOx exhibited higher trypanocidal activity towards epimastigotes and blood trypomastigotes than Et-NPOX, Bz and Nx

**Figure 2:**
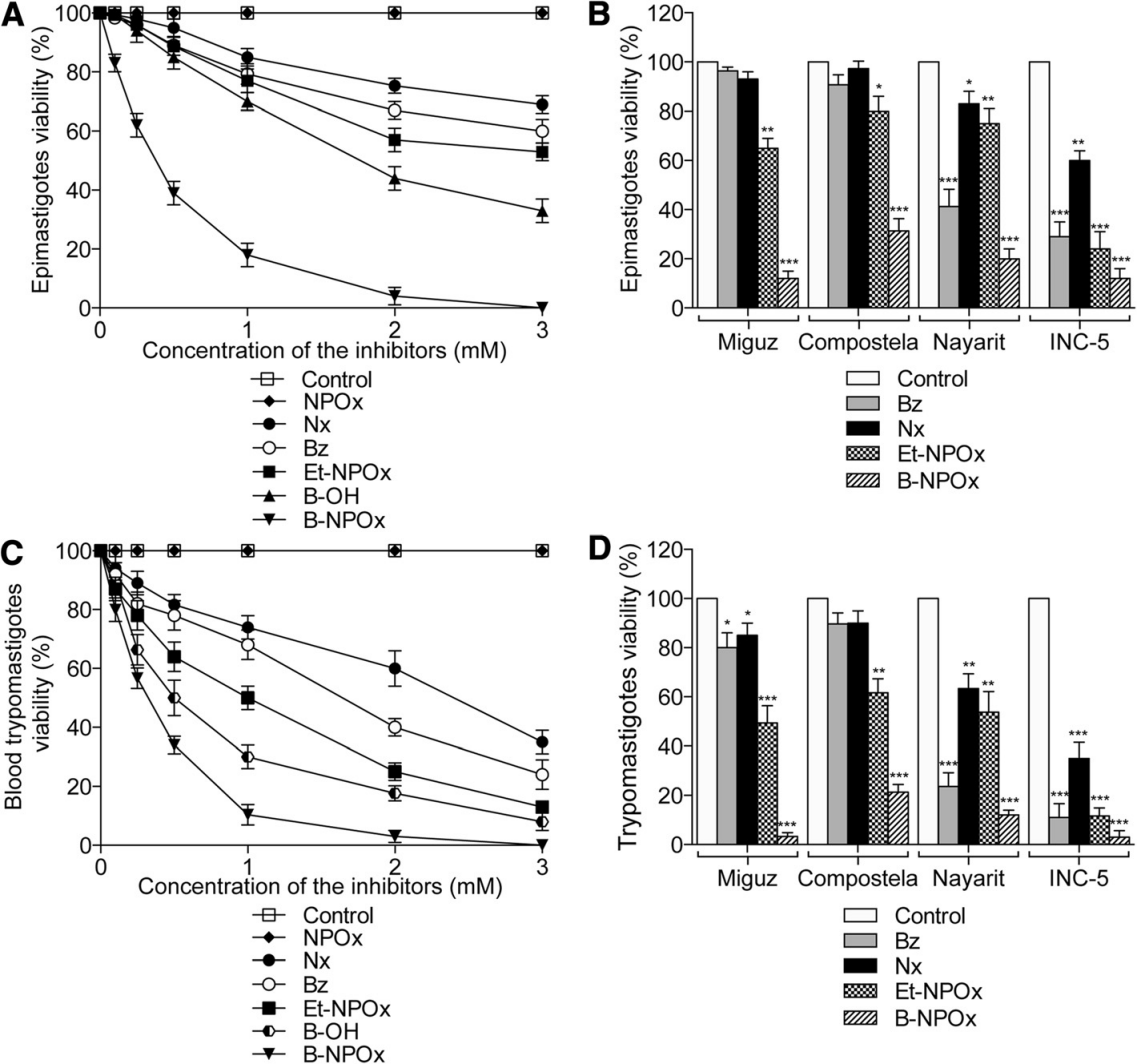
B-NPOx exhibited higher *in vitro* trypanocidal activity than Et-NPOX or the reference drugs Bz and Nx. **(A)** The viability of intact NINOA *T. cruzi* epimastigotes was evaluated using 1 × 10^6^ epimastigotes/mL after they had been treated with 0.1–3 mM B-NPOx, Et-NPOx, NPOx, Nx, Bz or benzyl alcohol (B-OH) every 10 min for 60 min at 28°C. The data shown correspond to an incubation period of 60 min. Viability was calculated as the number of live parasites, which was quantified using the trypan blue dye exclusion method. The control, which was set to 100%, represents the viability of the untreated epimastigotes. The results have been reported as the mean values ± the standard deviations for the viabilities of the epimastigotes from three independent experiments. **(B)** The effects of 1 mM B-NPOx, Et-NPOx, Bz and Nx towards the epimastigotes of the Miguz, Compostela, Nayarit and INC-5 strains of *T. cruzi* were evaluated under the conditions described in **(A). (C)** The viability of intact NINOA *T. cruzi* bloodstream trypomastigotes was evaluated based on the motility of 1 × 10^6^ bloodstream trypomastigotes/mL following their treatment with B-NPOx, Et-NPOx, NPOx, Bz or Nx (0.1–3 mM) in 1% DMSO for 24 h at 4°C. Blood containing 1 × 10^6^ trypomastigotes and 1% DMSO was used as a control. The results have been presented as the mean values ± the standard deviations for the viability of bloodstream trypomastigotes from three independent experiments. **(D)** The effects of 1 mM B-NPOx, Et-NPOx, Nx and Bz on 1 × 10^6^ bloodstream trypomastigotes/mL from the Miguz, Compostela, Nayarit and INC-5 strains of *T. cruzi* were evaluated under the conditions described in **(C)**. Data were analyzed in **(B)** and **(D)** with one-way ANOVA followed by Tukey’s multiple comparisons test. ***p ≤ 0.001, **p ≤ 0.01, *p ≤ 0.05, compared with the control.

B-NPOx penetrated into the epimastigotes of the NINOA strain of *T. cruzi*. When a suspension of the epimastigotes were incubated with 1 mM B-NPOx for 15, 30 and 45 min, the concentration of B-NPOx in the supernatants decreased by 9–15%, 30–34% and 40–46%, respectively. B-NPOx exhibited a higher trypanocidal activity towards the epimastigotes of the NINOA strain of *T. cruzi* than Et-NPOx, Bz and Nx at concentrations in the range of 0.1–1 mM (Figure 2A). The trypanocidal activity of B-NPOx was 4.4-fold higher than that of Bz at a concentration of 1 mM. Benzyl alcohol also exhibited trypanocidal activity by itself, which was higher than those of Et-NPOx, Bz and Nx (Figure 2A). The combination of benzyl alcohol and NPOx had a similar trypanocidal activity to that of benzyl alcohol alone. In contrast, ethyl alcohol had no trypanocidal activity (data not shown). B-NPOx also exhibited a higher trypanocidal activity towards the epimastigotes of the Nayarit (2.1-fold), INC-5 (2.4-fold), Compostela (2.9-fold) and Miguz (8.1-fold) strains of *T. cruzi* than Bz. Furthermore, the Miguz and Compostela strains of *T. cruzi* were resistant to Bz and Nx (compared with the untreated epimastigotes) (Figure 2B). The activity of B-NPOx was also higher than that of Et-NPOx towards the epimastigotes of the INC-5 (2-fold), Compostela (2.6-fold), Nayarit (3.75-fold), NINOA (4.3-fold Figure 2A) and Miguz (5.5-fold) strains of *T. cruzi* (Figure 2B). Notably, benzyl alcohol exhibited trypanocidal activity towards four of the *T. cruzi* strains tested in this study (data not shown).

B-NPOx exhibited the highest trypanocidal activity of all of the compounds tested towards blood trypomastigotes of the NINOA strain of *T. cruzi* at concentrations in the range of 0.1–1 mM. The activity of B-NPOx at 1 mM was 6.6-fold greater than that of Bz towards the NINOA strain (Figure 2C). Similar results were also obtained for the Nayarit (2-fold), INC-5 (3.7-fold), Compostela (4.2-fold) and Miguz (24-fold) strains of *T. cruzi* (Figure 2D). The activity of B-NPOx was also higher than that of Et-NPOx towards the Compostela (2.9-fold), INC-5 (3.9-fold), Nayarit (4.5-fold), NINOA (4.9-fold Figure 2C) and Miguz (14.8-fold) strains of *T. cruzi*. Blood trypomastigotes were found to be slightly more sensitive to the compounds than the epimastigotes, and the trypomastigotes of the Miguz strain were sensitive to Bz and Nx at p < 0.05 (Figure 2D). Benzyl alcohol also exhibited trypanocidal activity towards the blood trypomastigotes of all five of the different strains of *T. cruzi* tested in the current study, and the results for the NINOA strain are shown in Figure 2C.

### B-NPOx reduced parasitemia in infected mice more effectively than Et-NPOX, Bz or Nx

**Figure 3:**
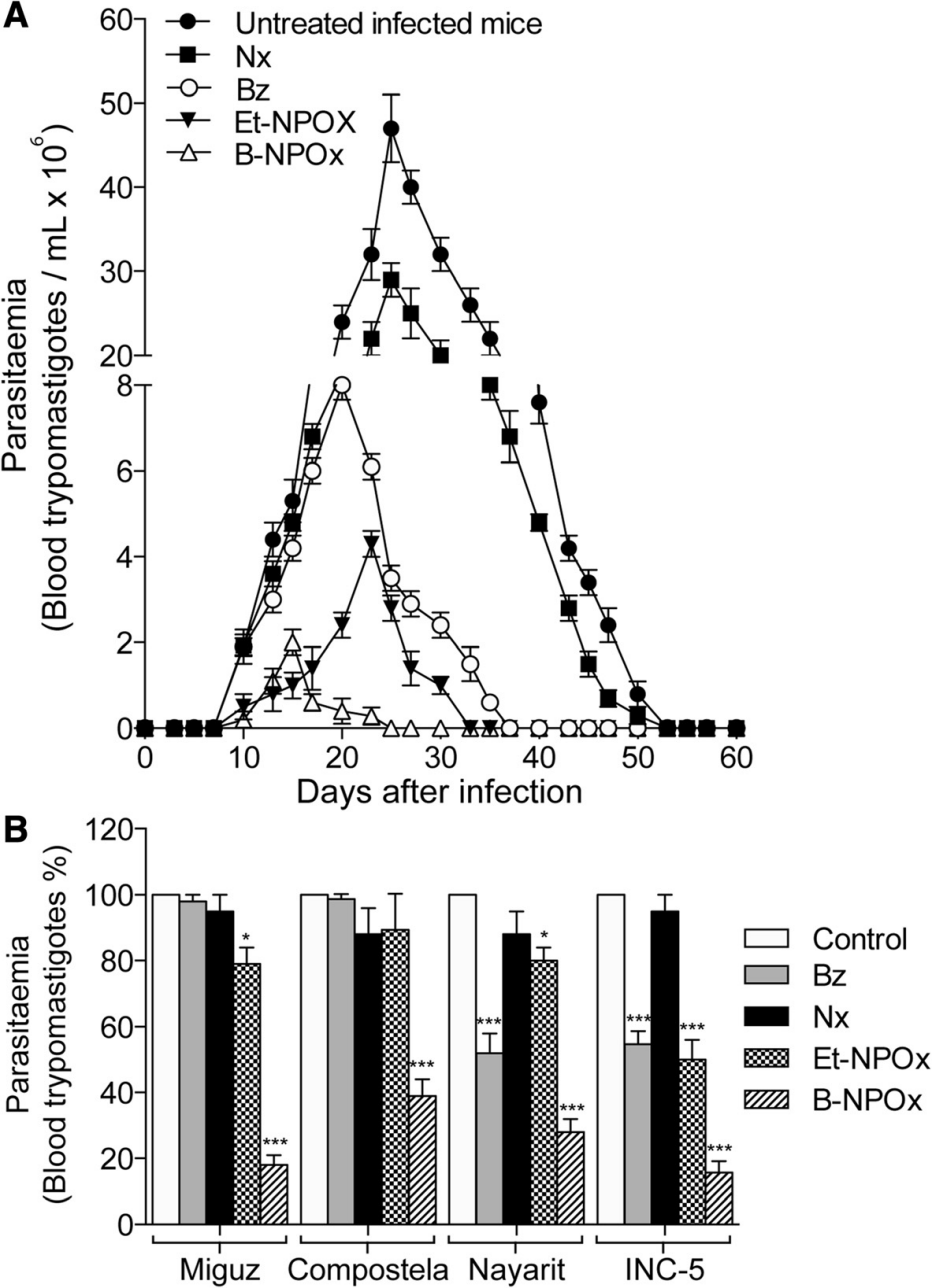
B-NPOx led to a greater reduction in the parasitemia in infected mice than Et-NPOX, Bz or Nx. **(A)** Parasitemia was evaluated according to the number of bloodstream trypomastigotes in mice infected with the NINOA strain of *T. cruzi* and subsequently treated with B-NPOx, Et-NPOx, Bz or Nx as indicated in the Methods section. A group of infected/untreated mice was used as a control group. The drugs were administered daily at 100 mg/kg over a period of 50 days. The first dose was given 24 h after the infection. The results have been presented as the mean values ± the standard deviations of the parasitemia from three different experiments. **(B)** The effects of B-NPOx, Et-NPOx, Bz and Nx towards the parasitemia in mice infected with the Miguz, Compostela, Nayarit and INC-5 strains of *T. cruzi* were evaluated under the conditions indicated in **(A)**. The percentage of blood trypomastigotes was calculated on the day of maximum parasitemia for all four of the different *T. cruzi* strains (i.e., 40–45 days after infection with the Miguz and Compostela strains, 15–20 days after infection with the Nayarit and INC-5 strains), and the parasitemia in the control (infected/untreated) group was set to 100% in each case. Data were analyzed with one-way ANOVA followed by Tukey’s multiple comparisons test. ***p ≤ 0.001, **p ≤ 0.01, *p ≤ 0.05, compared with the infected/untreated mice.

The parasitemia in mice infected with the NINOA strain of *T. cruzi* was detectable 10 days after infection, and reached its maximum value 25 days after infection before becoming non-detectable after 50 days of infection (Figure 3A). After 50 days of infection, the trypomastigotes infect new cells and become intracellular amastigotes [41]. The parasitemia induced by the Miguz and Compostela strains of *T. cruzi* was detectable 25–30 days after infection and reached its peak at 40–45 days, before becoming non-detectable after 55 days of infection. In contrast, the parasitemia induced by the Nayarit and INC-5 strains of *T. cruzi* was detectable 5 days after infection and reached its peak at 15–20 days before becoming non-detectable after 30 days of infection, as previously described [28].

B-NPOx led to a significant reduction in parasitemia in mice infected with the NINOA strain of *T. cruzi* compared with the infected/untreated mice (Figure 3A). B-NPOx caused the greatest reduction in parasitemia of all of the compounds tested in the current study, with the biggest reduction occurring between 10 and 20 days after infection (Figure 3A). For example, B-NPOx was found to be 15-fold more efficient than Bz at day 20. B-NPOx was also found to be more effective than Bz towards the Nayarit (1.9-fold), Compostela (2.5-fold), INC-5 (3.5-fold) and Miguz (5.4-fold) strains of *T. cruzi* (Figure 3B). It is noteworthy that the Miguz and Compostela strains of *T. cruzi* were resistant to treatment with Nx and Bz (Figure 3B). B-NPOx was also found to be more effective than Et-NPOx for treating the NINOA (2.2-fold, Figure 3A), Compostela (2.3-fold), Nayarit (2.9-fold), INC-5 (3.2-fold) and Miguz (4.5-fold) strains of *T. cruzi* (Figure 3B).

The erythrocytes were counted every time that the parasitemia was assessed, and no significant differences were found in the erythrocyte count in any of the mice groups infected with any of the five *T. cruzi* strains and treated with B-NPOx and Et-NPOx tested in the current study. The erythrocyte counts were always in the range of 6,000,000–10,000,000/mL in all of the mice, which is considered normal [42], and there was therefore no evidence to suggest that B-NPOx and Et-NPOx were exhibiting hemolytic effects. B-NPOx and Et-NPOx were not found to be cytotoxic towards Vero cells (85% or more cells were viable) at concentrations of up to 2 mM. The daily administration of Et-NPOx and B-NPOx for 50 days did not cause any overt tissue alterations in the mice. The LD_50_ values of B-NPOx and Et-NPOx were 2.00 and 2.32 g/kg of body weight, respectively, which indicated that these compounds are not toxic in mice [34].

### B-NPOx was more effective to reduce amastigote nests in infected mice than Et-NPOX, Bz and Nx

**Figure 4:**
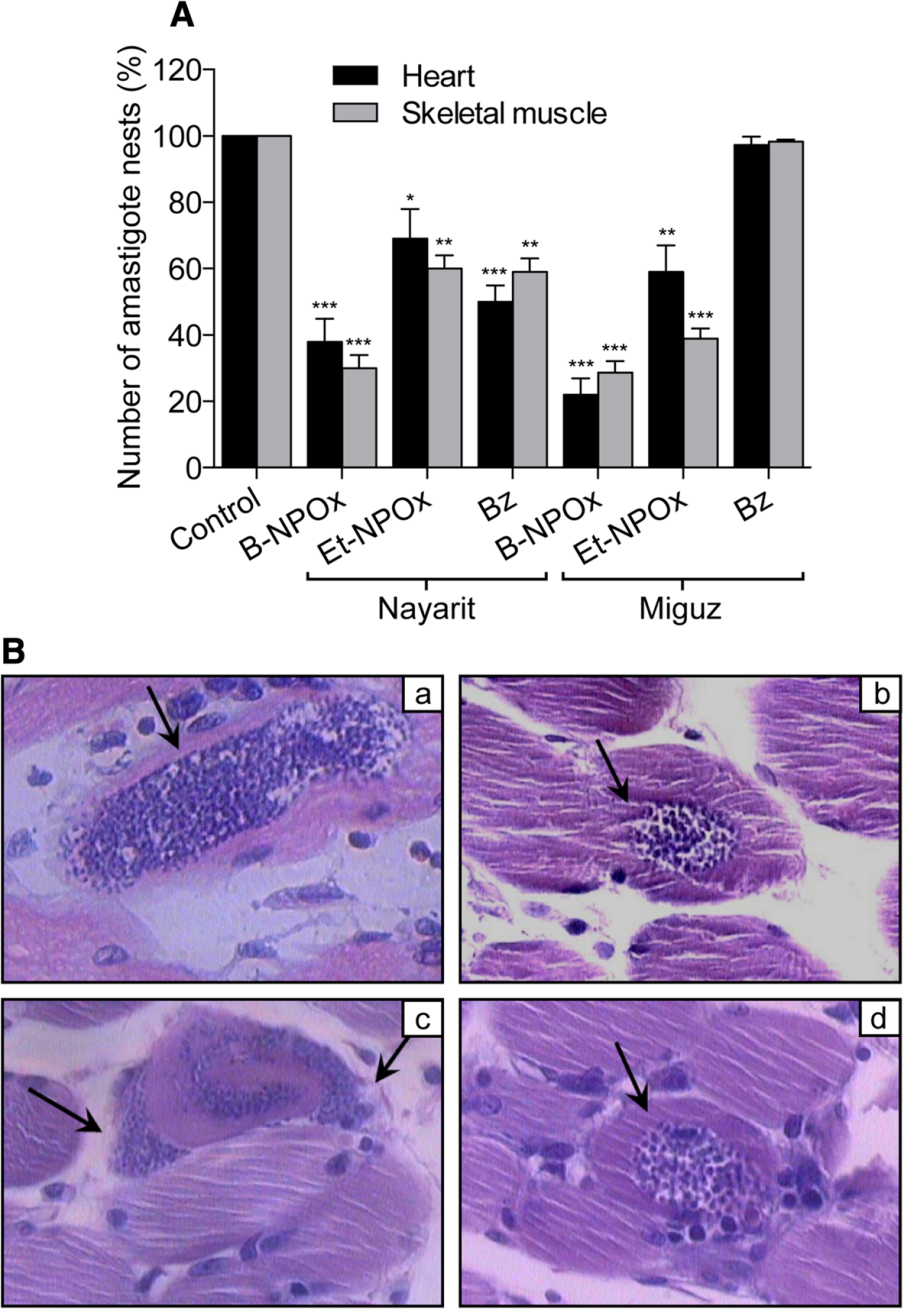
B-NPOx was more effective than Et-NPOX, Bz and Nx in terms of its ability to reduce amastigote nests in infected mice. **(A)** The effects of B-NPOx, Et-NPOx and Bz (daily dosing at 100 mg/kg for 30 or 50 days) on the amastigote nests were evaluated using tissue slices (3 μm thick) of the heart and skeletal muscles of mice infected with the Nayarit and Miguz strains of *T. cruzi* (these mice were the same than those studied in Figure 3). The tissue slices were stained with hematoxylin/eosin and analyzed by light microscopy. Fifty randomly selected microscopic fields from at least three mice were examined to quantify the number of amastigote nests. The mean number of amastigote nests in the infected/untreated group was set as 100%. Data were analyzed with one-way ANOVA followed by Tukey’s multiple comparisons test. ***p ≤ 0.001, **p ≤ 0.01, *p ≤ 0.05, compared with the infected/untreated mice. **(B)** Histological studies of the myocardium and skeletal muscle of mice infected with the NINOA strain of *T. cruzi*. Samples of the myocardium (a, b) and skeletal muscle (c, d) from infected/untreated mice (a, c) or of mice (b, d) treated with B-NPOx at 100 mg/kg over 50 days. Tissue slices stained with hematoxylin/eosin were analyzed at 40× magnification. The arrows indicate the amastigote nests. The images are representative of three different experiments.

The number of amastigote nests found in the heart and skeletal muscles of the mice treated with B-NPOx was significantly reduced compared with the infected/untreated mice, as shown for the Nayarit (2.6-fold) and Miguz (4.6-fold) strains of *T. cruzi* in Figure 4A. This effect was also higher than that found in mice treated with Et-NPOx or Bz. It is noteworthy that the Miguz strain of *T. cruzi* was resistant to Bz (Figure 4A). There were no significant differences in the number of amastigote nests found in the heart and skeletal muscles of any one group. Similar results were also found with the NINOA (2.1-fold), INC-5 (3.4-fold) and Compostela (4.1-fold) strains (data not shown). The effects of Nx were lower than those of Bz (data not shown). Histological analysis of the myocardium and the skeletal muscles of mice infected with the NINOA and INC-5 strains that had been treated with B-NPOx showed a significantly reduction in the size of the amastigote nests, as shown for the NINOA strain in Figure 4B and without any inflammatory infiltrate (Figure 4B).

## Discussion

The aim of the current study was to develop an efficient prodrug for the treatment of Chagas disease using a murine model of the disease. With this in mind, we designed and synthesized B-NPOx, and subsequently evaluated its activity towards five different strains of *T. cruzi* relative to those of Et-NPOx and the reference drugs Bz and Nx. In our previous study, we demonstrated that NPOx is an effective and selective inhibitor of HADH-isozyme II of *T. cruzi* [24]. Unfortunately, however, NPOx is a very polar molecule and can therefore not penetrate intact epimastigotes, because cellular membranes are hydrophobic barriers that block the diffusion of polar substances. In contrast, the hydrophobic compound Et-NPOx has been reported to enter intact epimastigotes and exhibit trypanocidal activity [24,27].

**Figure 5:**
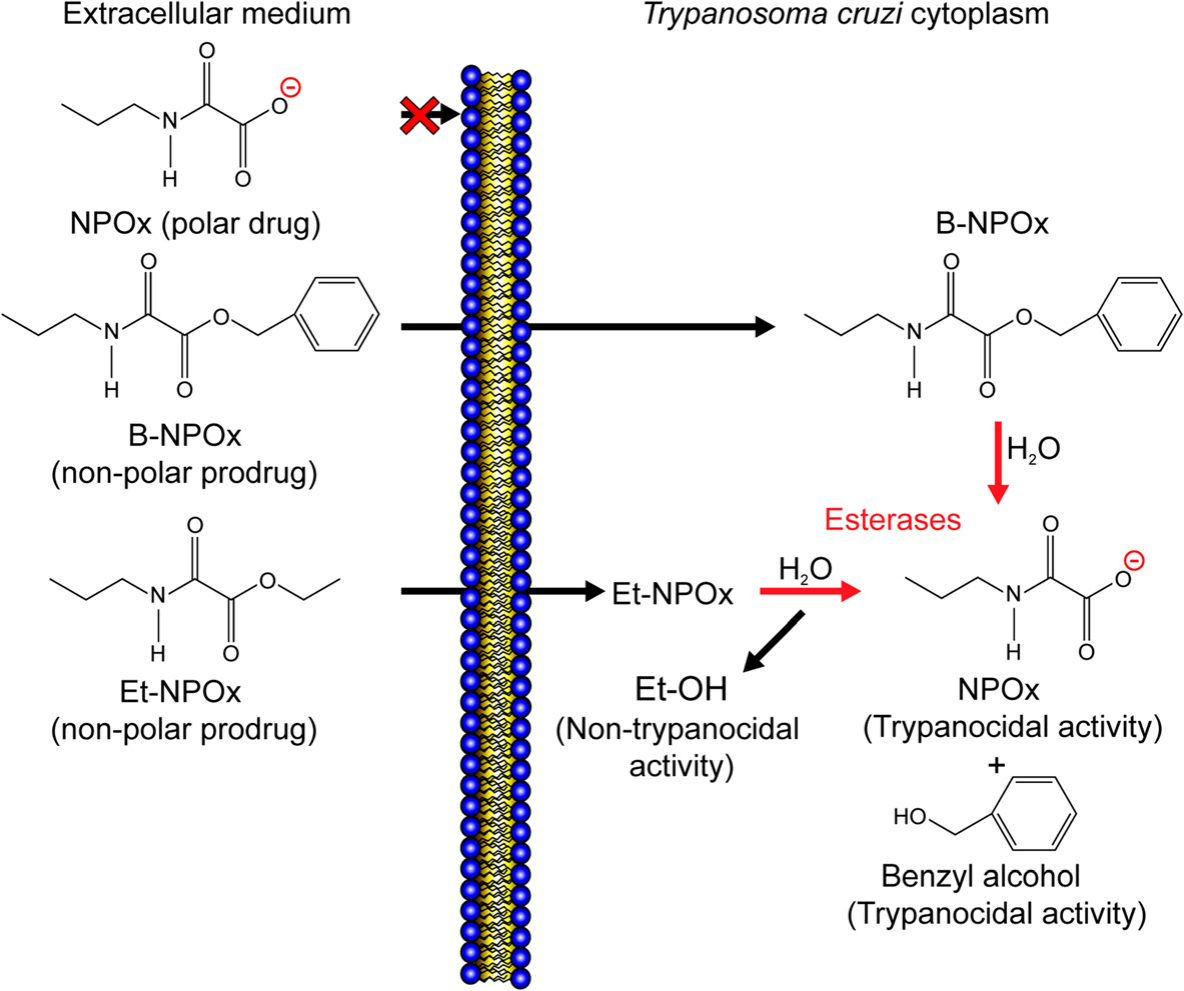
Proposed mechanism for the trypanocidal activity of the benzyl and ethyl esters of NPOx. B-NPOx and Et-NPOx would diffuse through the cellular membrane of *T. cruzi*, where they would be hydrolyzed by intracellular aliphatic and aromatic carboxyl esterases to give NPOx, which is a selective and competitive inhibitor of HADH-isozyme II. Et-NPOx would also give the inactive ethyl alcohol, whereas B-NPOx would give benzyl alcohol, which is a well know antimicrobial agent that also exhibits trypanocidal activity (as we demonstrated here). B-NPOx would therefore exhibit dual trypanocidal activity resulting from NPOx and benzyl alcohol. Furthermore, the lipophilicity of B-NPOx was found to be 1.76-fold greater than that of Et-NPOx, which would allow it to penetrate more effectively into *T. cruzi* than Et-NPOx to give a higher level of trypanocidal activity.

The results of the current study have shown that B-NPOx behaves in the same way as Et-NPOx towards purified HADH-isozyme II, in the sense that it did not inhibit its activity, but B-NPOx did exhibit significant inhibitory activity towards *T. cruzi* homogenates, because the homogenates contain aromatic and aliphatic non-specific carboxyl esterases [30,31] that can release the NPOx inhibitor. In the presence of N-ethylmaleimide, which is an inhibitor of the carboxylesterases [30,31], the inhibitory activities of B-NPOx and Et-NPOx towards the *T. cruzi* homogenates were reduced to less than 10% of their original values. Notably, N-ethylmaleimide did not inhibit the activity of purified HADH-isozyme II. These findings strongly support the mechanism that we proposed for the activity of the esters of NPOx. Briefly, following the penetration of the hydrophobic prodrug B-NPOx or Et-NPOx into *T. cruzi* and its subsequent hydrolysis to release the NPOx inhibitor (Figure 5), the inhibition of HADH-isozyme II by NPOx would block the oxidation of NADH, which would lead to a significant decrease in the energy supply that would ultimately kill the *T. cruzi* parasites.

The lipophilicity of B-NPOx was measured in terms of its partition coefficient LogP (i.e., octanol:water log partition coefficient), which was determined using the CAS System of the American Chemical Society [43,44]. The LogP values for NPOx and Et-NPOx were determined to be 0.487 and 1.52, respectively. Given that B-NPOX is a novel compound, its LogP value has not been published in the literature, although this number can be calculated by the sum of the hydrophobic contributions of the “parent” molecule and its corresponding substituents [43]. For B-NPOX, the “parent” molecule would be the methyl ester of N-propyl oxamate (Me-NPOx) (LogP = 1.013) minus one proton (0.225). The substituent in this case would be benzene minus one proton (LogP = 1.90), and so the sum of these values would give a calculated LogP value of 2.688 for B-NPOX. Based on these results, the lipophilicity of B-NPOX would be 1.77-fold higher than that of Et-NPOX.

The high lipophilicity of B-NPOx would allow it penetrate more readily through the *T. cruzi* membranes, which would lead to higher concentrations of B-NPOx inside the *T. cruzi* parasites compared with Et-NPOx. The presence of higher concentrations of B-NPOx inside the parasite would consequently lead to higher levels of NPOx following the hydrolysis of the prodrug, which would inhibit the activity of HADH-isozyme II. The high trypanocidal activity of B-NPOx can also be attributed to the effect of the benzyl alcohol, which is released from B-NPOx during its hydrolysis inside the parasites. As a result of the high lipophilicity of B-NPOx and the dual inhibitory effects of NPOx and benzyl alcohol, this prodrug can deliver a higher level of trypanocidal activity than Et-NPOx or Bz. The activities of B-NPOx towards the epimastigotes, trypomastigotes and parasitaemia of the five different strains of *T. cruzi* tested in the current study were 2- to 5.5-fold, 2.9- to 14.8-fold and 2.2- to 4.5-fold higher than those of Et-NPOx, respectively. Furthermore, the activities of B-NPOx towards the epimastigotes, trypomastigotes and parasitaemia of the five different strains of *T. cruzi* tested in the current study were 2.1 to 8.1-fold, 2- to 24-fold and 1.9- to 15-fold greater than those of Bz, respectively. The effects of Nx were always lower than those of Bz. It is noteworthy that B-NPOx exhibited trypanocidal activity and reduced the parasitemia produced by Miguz and Compostela strains of *T. cruzi*, which were resistant to treatment with Bz and Nx. Taken together, these results suggest that B-NPOx could be used as a bi-potential prodrug for the treatment of Chagas disease.

The antimicrobial activity of benzyl alcohol (growth inhibition and bacterial killing) can be explained by its inhibition of several membrane proteins and its ability to cause changes in cell membrane fluidity [45,46]. The trypanocidal activity of benzyl alcohol, which has been reported for the first time in this study, could also be explained by similar mechanisms.

These findings demonstrate that B-NPOx was the most effective of all of the compounds tested in the current study against the infective (blood trypomastigotes) and the reproductive (amastigotes) forms of *T. cruzi* in mice. Blood trypomastigotes have a vigorous motility, which allows for their rapid extravasation and dissemination within the host, where they invade fibroblasts, macrophages and epithelial cells. Once in these cells, trypomastigotes can undergo differentiation into amastigotes. Mobile trypomastigotes subsequently emerge from amastigote nests, leading to the rupture of the infected cells during the chronic stage of the disease [5]. The dual effect of B-NPOx against the infective and the reproductive forms of *T. cruzi* provides strong evidence in support of the use of B-NPOx as a potent therapeutic prodrug for the treatment of Chagas disease.

In addition to its ability to inhibit the activity of HADH-isozyme II, treatment with NPOx also leads to an increase in the concentrations of the HADH-isozyme II substrates α-ketocaproate and α-ketoisocaproate, which are derived from leucine and isoleucine through transamination reactions. It is therefore possible that NPOx also affects the metabolism of these amino acids and that these changes could play a critical role in the killing of *T. cruzi*. It is expected that the long evolutionary distance between trypanosomatids and their mammalian hosts would allow for the selective inhibition of the enzymes belonging to the trypanosomatids without affecting the host enzymes [20].

B-NPOx was not found to be cytotoxic and there was no evidence of hemolytic damage or overt tissue alteration in mice treated with B-NPOx for 50 days. The doses of B-NPOx administered to the mice (100 mg/kg of body weight) were 20-fold lower than that its LD_50_ value. B-NPOx is made up of two polar compounds that can be eliminated from the host (humans or animals) through phase II metabolism processes (e.g., esterification). In contrast, other drugs commonly used for the treatment of Chagas disease, such as Nx, Bz and fexinidazole (which is commonly used in human African trypanosomiasis and in experimental models of Chagas disease [1]), are highly toxic non-polar molecules that are difficult to eliminate [14]. These properties lend further support to the idea that B-NPOx could potentially be used as a therapeutic prodrug for the treatment of Chagas disease.

The combination of two or more active compounds in one drug preparation (multi-potential drug) has been described previous as a general strategy to increase the therapeutic efficacy of the individual components [32,47,48]. B-NPOx is a bi-potential prodrug, and the strategy reported in the current study could be used to introduce other antimicrobial agents, besides benzyl alcohol, into *T. cruzi*. We are currently studying the effects of B-NPOx on the promastigotes and amastigotes of *Leishmania* (another member of the Trypanosomatidae family), which probably contain an aromatic α-hydroxy acid dehydrogenase, as well as several other α-hydroxy acid dehydrogenases [49].

Additional pharmacological and toxicological studies of the effects of B-NPOx on other animals are required to fully evaluate its usefulness as a therapeutic prodrug for the treatment of Chagas disease. The results presented in the current study could have a significant impact on human health because there are currently no drugs available for the effective treatment of Chagas disease. This disease affects millions of people in Latin America, and so the search for new drugs against *T. cruzi* is of utmost importance and must be continued.

## Conclusions

B-NPOx exhibited a higher trypanocidal effects than Et-NPOx both *in vitro* (2- to 14.8-fold) towards epimastigotes and trypomastigotes and *in vivo* (2.2- to 4.5-fold) towards the blood trypomastigotes and tissues of mice infected with the five different strains of *T. cruzi*. B-NPOx also exhibited greater activities *in vitro* (2- to 24-fold) and *in vivo* (1.9- to 15-fold) compared with the Bz and Nx. The higher lipophilicity of B-NPOx compared with Et-NPOx (1.77-fold) could be responsible for its better penetration into *T. cruzi*, where its enzymatic cleavage would result in the release of the NPOx inhibitor and benzyl alcohol, which both exhibit sufficiently high levels of trypanocidal activity to kill *T. cruzi*. Taken together with its low toxicity, these results therefore indicate that B-NPOx could be used as a potent therapeutic prodrug for the treatment of Chagas disease.

## Abbreviations

HADH: α-hydroxy acid dehydrogenase
NAOx: N-allyl oxamate
NIPOx: N-isopropyl oxamate
NPOx: N-propyl oxamate
Et-NPOx: Ethyl ester of NPOx
B-NPOx: Benzyl ester of NPOx
Bz: Benznidazole
Nx: Nifurtimox
